# Association of early acetaminophen administration with mortality in surgical intensive care patients: a retrospective cohort study

**DOI:** 10.3389/fphar.2025.1588978

**Published:** 2025-08-25

**Authors:** Jiajing Li, Yihao Li, Min Zhong, Hanli Chen, Ziyang Wang, Jianwei Wu, Yongyong Shi

**Affiliations:** ^1^ Department of Anesthesiology, The Second Affiliated Hospital of Guangzhou University of Chinese Medicine, Guangzhou, China; ^2^ Department of Anesthesiology, The Second Affiliated Hospital of Guangzhou Medical University, Guangzhou, China

**Keywords:** acetaminophen, critical illness, mortality, surgical intensive care unit, oxidative stress

## Abstract

**Background:**

Acetaminophen, a widely used analgesic, has drawn attention for its potential to reduce oxidative stress through inhibiting lipid peroxidation and scavenging free radicals. Emerging evidence indicates that early acetaminophen administration might improve survival outcomes in surgical intensive care unit (SICU) patients. This study aims to explore the relationship between early acetaminophen use and mortality in this patient population.

**Methods:**

We conducted a retrospective cohort analysis of adult SICU patients using the Medical Information Mart for Intensive Care (MIMIC-IV) database, which contains data on patients admitted between 2008 and 2019. The intervention cohort received acetaminophen within 48 h of ICU admission, while controls received no early acetaminophen. The primary endpoint was in-hospital mortality. We implemented 1:1 propensity score matching (PSM) to minimize selection bias and balance baseline characteristics between cohorts. Multivariable Cox regression models adjusted for residual confounding.

**Results:**

From 5,474 eligible patients, we generated balanced cohorts of 2,740 matched pairs. The acetaminophen group demonstrated significantly lower in-hospital mortality (11.4% vs 14.4%; adjusted HR 0.75, 95% CI 0.62–0.89, *P* = 0.001) compared to controls. This mortality reduction persisted consistently through sensitivity analyses and subgroup evaluations. Notably, early acetaminophen administration associated with improved survival metrics across multiple timepoints: ICU mortality (adjusted HR 0.63, 0.48–0.82; *P* = 0.001), 90-day mortality (adjusted HR 0.78, 0.66–0.92; *P* = 0.004), and 180-day mortality (adjusted HR 0.78, 0.67–0.92; *P* = 0.002). While no significant differences were observed in ICU or hospital length of stay between groups, early acetaminophen administration was linked to longer ICU-free days through day 28 (*β* = 0.71; *P* = 0.022) and longer vasopressor-free days through day 28 (*β* = 0.77; *P* = 0.012).

**Conclusion:**

Early acetaminophen administration demonstrates independent associations with reduced in-hospital mortality and improved secondary clinical outcomes in surgical ICU populations. These findings highlight the need for prospective trials to confirm therapeutic efficacy and establish causal inference for this readily available pharmacologic intervention.

## Introduction

Despite progress in intensive care units, the prognosis for surgical intensive care unit (SICU) patients remains poor, particularly in low - income countries, where mortality and morbidity rates are high (Endeshaw et al., 2023). Comprehending the determinants of adverse outcomes and identifying effective treatment strategies are essential for enhancing patient survival and quality of life; pinpointing modifiable risk factors and therapeutic interventions remains a primary focus in SICU - related research and clinical practice.

SICU patients often face significant oxidative stress, marked by excessive free radical production, which can lead to tissue damage, organ dysfunction, and even death. (Karapetsa et al., 2013; Smith et al., 2021). In response to oxidative stress, various pharmacologic interventions have been explored. Among these, acetaminophen (also known as paracetamol), a widely used antipyretic and analgesic, has drawn clinical attention for its potential antioxidant properties. Evidence suggests that acetaminophen may be beneficial for critically ill patients. For instance, Sun et al. observed that acetaminophen use was associated with reduced mortality in critically ill septic patients (Sun et al., 2024). Early ICU administration of acetaminophen has also been noted to protect kidney function (Janz et al., 2015). However, there are concerns about the risk of significant hypotension with its use in critical care, which could potentially worsen patient outcomes (Kelly et al., 2016). Additionally, acetaminophen-induced liver toxicity remains a leading cause of acute liver failure globally (Akakpo et al., 2020). Despite its widespread use in critical care, as shown by a large-scale observational study where 64% of ICU patients received acetaminophen during hospitalization (Suzuki et al., 2015), evidence regarding its impact on ICU patient outcomes, particularly in the SICU, is still limited. The relationship between acetaminophen use, its timing, and mortality in surgical intensive care patients is not yet well understood and warrants further investigation.

Therefore, we aimed to examine the association between early acetaminophen administration and mortality in surgical intensive care patients. We hypothesized that early acetaminophen use might reduce mortality and improve prognoses in these patients.

## Methods

### Data source

We conducted a retrospective cohort study based on a large US-based database called the Medical Information Mart for Intensive Care IV (MIMIC-IV). The MIMIC-IV (v2.2) database contains comprehensive and high-quality data of well-defined and characterized ICU patients admitted to ICUs at the Beth Israel Deaconess Medical Center between 2008 and 2019 (Johnson et al., 2023). The Institutional Review Board at Beth Israel Deaconess Medical Center approved the sharing of research resources and granted a waiver of informed consent. Author YL accessed the database and performed data extraction (certification number 46484149). The manuscript aligns with the Strengthening the Reporting of Observational Studies in Epidemiology (STROBE) statement (von Elm et al., 2007).

### Study population

**FIGURE 1:**
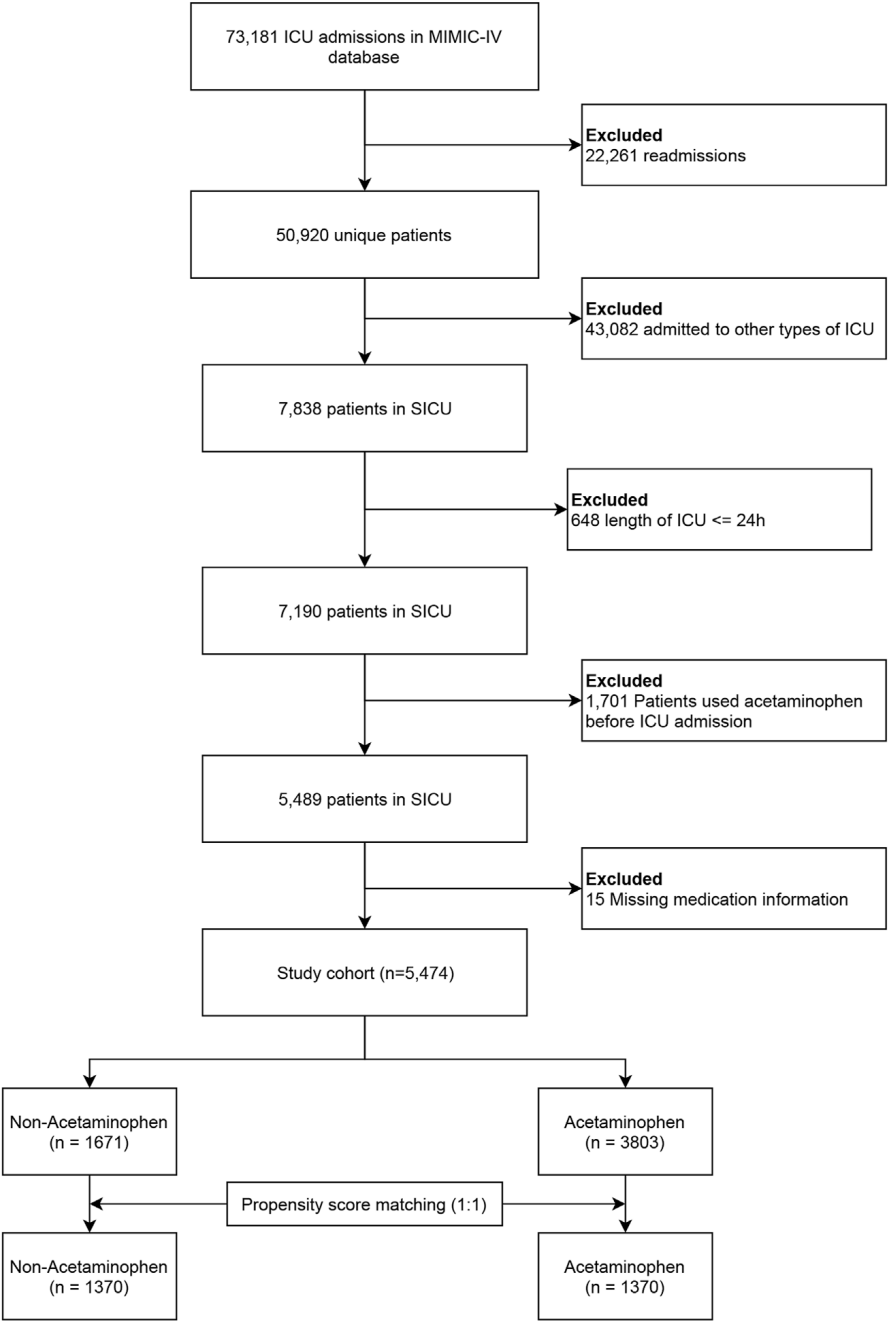
Study flowchart. Abbreviations: MIMIC, the Medical Information Mart for Intensive Care; SICU, surgical intensive care unit.

This study included adult critically ill patients admitted to the SICU. We excluded patients under 18, those with ICU stays under 24 h, those who had taken acetaminophen before ICU admission, and those with incomplete records of acetaminophen administration times or routes. For patients with multiple ICU admissions, only the first admission was considered. Figure 1 shows the patient selection process.

### Exposure and outcomes

The exposure was defined as receiving at least one dose of acetaminophen in any form within 48 h after ICU admission. Our definition of the 48 - hour time frame for early postoperative acetaminophen exposure was derived from two preceding studies. Their research suggested that receiving acetaminophen within 48 h of admission was linked to a more favorable prognosis (Suzuki et al., 2015; Xiong et al., 2023). Acetaminophen use was extracted from the “prescriptions” table in the MIMIC database. Patients who received acetaminophen solely after 48 h were deemed unexposed in our primary analysis. The primary outcome was in-hospital mortality. Secondary outcomes included ICU mortality, 90-day mortality, 180-day mortality, durations of ICU and hospital stay, ICU-free days to day 28, and vasopressor-free days to day 28. ICU-free days to day 28 were calculated as 28 minus the number of days spent in the ICU over a period of 28 days (Fowler et al., 2019). Vasopressor-free days to day 28 were determined by subtracting the number of days of vasopressor support within the first 28 days after ICU admission from 28; patients who died within 28 days were assigned 0 vasopressor - free days.

### Data extraction

Data extraction was performed using Structured Query Language (SQL). The SQL script codes were obtained from the GitHub website (https://github.com/MIT-LCP/mimic-iv). Patient characteristics, including age, sex, race, weight, and elective surgery status were collected. We extracted information on comorbidities, such as prior myocardial infarction, congestive heart failure, cerebrovascular disease, chronic pulmonary disease, renal disease, liver disease, hypertension, diabetes, sepsis, and malignant cancer. We extracted following covariates within the first 24 h after ICU admission, including treatment (vasopressor and renal replacement therapy), severity of illness (simplified acute physiology score [SAPS] II, charlson comorbidity index, sequential organ failure assessment [SOFA], oxford acute severity of illness score [OASIS]), vital sign (mean blood pressure, respiratory rate, temperature, heart rate), and laboratory findings (white blood cell [WBC], platelets, hemoglobin, glucose, creatinine). Variables with missing data exceeding 5% were excluded; multiple imputations were performed to address the missing data.

### Statistical analysis

Given the retrospective nature of the study, no preliminary statistical plan was established. Statistical power calculations were not executed, and the sample size was determined by the accessible data within the database. Patients who received their first dose of acetaminophen within 48 h after ICU admission were assigned to the ACE group, while the rest of the patients comprised the non-ACE group. Values are reported as means (standard deviations) or medians [interquartile ranges (IQRs)] for continuous variables depending on the normality of the distribution, and as total numbers and percentages for categorical variables. For group comparisons, the X^2^ test or Fisher’s exact test were employed for categorical variables, while the Student’s t-test or Mann-Whitney U test were used for continuous variables, as suitable.

Multivariable Cox models were developed to calculate the hazard ratio (HR) and 95% confidence interval (CI) for the primary outcome (in-hospital mortality). Covariate selection was based on prior studies (Suzuki et al., 2015; Xiong et al., 2023; Liu et al., 2024) and clinical expertise. Variables in Table 1 were treated as potential confounders and included in the model. Subgroup analyses were conducted based on relevant covariates—age, sex, and histories of myocardial infarction, cerebrovascular disease, renal disease, diabetes, sepsis, elective surgery, congestive heart failure, chronic pulmonary disease, liver disease, hypertension, and malignancy—to reduce the impact of survival bias. Propensity score matching (PSM) and inverse probability of treatment weighing (IPTW) were employed to adjust covariates and reinforce the reliability of our results (Zhang, 2017; Grafféo et al., 2019). A multivariate logistic regression determined propensity scores for acetaminophen exposure. We applied one-to-one nearest neighbor matching within a caliper of 0.2. IPTW models used these propensity scores as weights. The variables in Table 1 were chosen to compute the propensity score. Standardized mean differences (SMDs) assessed the effectiveness of PSM and IPTW, with <0.1 indicating acceptability. Cox regression was subsequently conducted on both matched and weighted cohorts.

**TABLE 1:** Characteristics before and after propensity score matching.

Variables	Before propensity score matching			After propensity score matching		
	Non-ACE group (n = 1,671)	ACE group (n = 3,803)	SMD	Non-ACE group (n = 1,370)	ACE group (n = 1,370)	SMD
Baseline characteristics						
Age, years	63.8 ± 16.4	63.2 ± 17.5	0.0403	64.4 ± 17.1	65.2 ± 16.9	0.0485
Sex, female	727 (43.5)	1921 (50.5)	0.1407	622 (45.4)	633 (46.2)	0.0161
Race			0.0322			0.0213
White	1,159 (69.4)	2,690 (70.7)		947 (69.1)	957 (69.9)	
Black	155 (9.3)	326 (8.6)		119 (8.7)	121 (8.8)	
Others/unknown	357 (21.4)	787 (20.7)		304 (22.2)	292 (21.3)	
Weight, kg	80.9 ± 23.8	80.7 ± 33.4	0.0044	80.6 ± 24.3	80.9 ± 28.5	0.0125
Admission following elective surgery	25 (1.5)	158 (4.2)	0.1610	24 (1.8)	26 (1.9)	0.0109
Comorbidities						
Prior myocardial infarction	142 (8.5)	278 (7.3)	0.0440	122 (8.9)	121 (8.8)	0.0026
Congestive heart failure	263 (15.7)	415 (10.9)	0.1424	221 (16.1)	221 (16.1)	<0.0001
Cerebrovascular disease	383 (22.9)	1,608 (42.3)	0.4222	372 (27.2)	386 (28.2)	0.0228
Chronic pulmonary disease	365 (21.8)	715 (18.8)	0.0757	300 (21.9)	306 (22.3)	0.0106
Renal disease	260 (15.6)	418 (11)	0.1349	213 (15.5)	217 (15.8)	0.008
Liver disease	482 (28.8)	256 (6.7)	0.6041	222 (16.2)	227 (16.6)	0.0099
Hypertension	763 (45.7)	1805 (47.5)	0.0361	646 (47.2)	639 (46.6)	0.0102
Diabetes	440 (26.3)	811 (21.3)	0.1177	354 (25.8)	353 (25.8)	0.0017
Sepsis	917 (54.9)	1,422 (37.4)	0.3563	670 (48.9)	677 (49.4)	0.0102
Malignant cancer	317 (19)	473 (12.4)	0.1803	208 (15.2)	215 (15.7)	0.0141
Severity of illness						
SAPS II	36.8 ± 14.6	30.7 ± 12.7	0.4491	34.7 ± 13.5	34.7 ± 13.9	0.0013
Charlson comorbidity index	6.0 (4.0, 8.0)	5.0 (3.0, 7.0)	0.2199	5.0 (3.0, 7.0)	5.0 (4.0, 7.0)	0.0369
SOFA	1.0 (0.0, 3.0)	0.0 (0.0, 1.0)	0.4776	1.0 (0.0, 2.0)	1.0 (0.0, 2.0)	0.0108
OASIS	33.1 ± 9.7	29.4 ± 9.3	0.3839	32.2 ± 9.5	32.1 ± 9.6	0.0105
Treatment						
Vasopressor	455 (27.2)	517 (13.6)	0.3432	303 (22.1)	287 (20.9)	0.0284
Renal replacement therapy	84 (5)	73 (1.9)	0.1703	50 (3.6)	52 (3.8)	0.0077
Vital sign						
Mean blood pressure, mmHg	60.4 ± 15.1	62.5 ± 13.2	0.1500	60.8 ± 14.9	61.1 ± 12.9	0.0233
Respiratory rate, breaths/min	27.1 ± 6.0	26.6 ± 5.9	0.0700	27.0 ± 5.8	26.8 ± 5.9	0.0279
Temperature, °C	37.3 ± 0.6	37.5 ± 0.7	0.2370	37.4 ± 0.6	37.4 ± 0.7	0.0032
Heart rate, beats/min	103.4 ± 19.9	100.5 ± 19.1	0.1505	103.2 ± 19.7	102.7 ± 20.7	0.0205
Laboratory findings						
WBC, K/µL	9.5 ± 5.1	10.7 ± 7.7	0.1841	9.9 ± 5.0	9.8 ± 4.6	0.0075
Platelets, K/µL	182.3 ± 111.0	212.3 ± 86.5	0.3024	200.1 ± 109.5	203.4 ± 92.5	0.0327
Hemoglobin, g/dL	10.6 ± 2.3	11.3 ± 2.1	0.3536	10.8 ± 2.2	10.9 ± 2.2	0.0234
Glucose, mg/dL	122.0 ± 43.0	123.6 ± 36.3	0.0402	121.8 ± 42.0	121.9 ± 37.6	0.0019
Creatinine, mg/dl	1.1 (0.8, 1.6)	0.9 (0.7, 1.2)	0.2390	1.0 (0.8, 1.5)	1.0 (0.8, 1.4)	0.0216

Note: Continuous variables are presented as mean (standard deviation) or median (interquartile), and categorical variables as count (%).

Abbreviations: ACE, acetaminophen; SMD, standardized mean difference; SAPS, simplified acute physiology score; SOFA, sequential organ failure assessment; OASIS, oxford acute severity of illness score; WBC, white blood cell.

The relationship between acetaminophen exposure and secondary outcomes was examined using matched cohorts. Multivariable Cox regression assessed the association with mortality (ICU, 90-day, and 180-day). Multivariable linear models were used to estimate adjusted coefficients and 95% CIs for the durations of ICU and hospital stay, ICU - free days to day 28, and vasopressor - free days to day 28.

A two-tailed test was conducted, with *p* < 0.05 deemed statistically significant. All analyses were conducted using R Statistical Software (Version 4.2.2, http://www.R-project.org, The R Foundation) and Free Statistics analysis platform (Version 1.8, Beijing, China).

### Sensitivity analysis

To evaluate the impact of missing data and multiple imputation on the primary outcome, multivariable Cox regression analyses were performed pre- and post-imputation.

## Results

### Baseline characteristics

Figure 1 shows the patient selection process. After strict screening using predefined criteria, 5,474 patients were included. The matched cohort comprised 2,740 patients; 1,370 per group. Fewer than 2% of relevant variables were missing (Supplementary Table S1). Of the entire cohort, 69.5% (3,803 patients) used acetaminophen within 48 h post-ICU admission. Baseline characteristics, categorized by acetaminophen use, are in Table 1.

In the entire cohort, ACE group had a higher prevalence of females, emergency surgery admissions, and histories of cerebrovascular disease and hypertension, while there were fewer histories of myocardial infarction, congestive heart failure, chronic pulmonary disease, renal and liver diseases, diabetes, sepsis, and malignant cancer. PSM resulted in improved variable balance, with an absolute SMD<0.10.

Table 2 shows the crude outcomes. The overall in-hospital mortality was 11.5% (631/5,474) of the entire cohort. Patients in the ACE group showed lower in-hospital, ICU, 90-day, and 180-day mortalities, shorter ICU and hospital stays, and longer ICU-free days to day 28 and vasopressor-free days to day 28.

**TABLE 2:** Crude outcomes before and after propensity score matching.

Outcomes	Before propensity score matching			After propensity score matching		
	Non-ACE group (n = 1,671)	ACE group (n = 3,803)	*P* Value	Non-ACE group (n = 1,370)	ACE group (n = 1,370)	*P* Value
Primary outcome						
In-hospital mortality	267 (16)	364 (9.6)	<0.001	197 (14.4)	156 (11.4)	0.019
Secondary outcomes						
CU mortality	194 (11.6)	235 (6.2)	<0.001	146 (10.7)	96 (7)	<0.001
90-day mortality	390 (23.3)	585 (15.4)	<0.001	302 (22)	256 (18.7)	0.029
180-day mortality	448 (26.8)	697 (18.3)	<0.001	346 (25.3)	301 (22)	0.043
Length of ICU stay, days	2.9 (1.9, 5.5)	2.7 (1.8, 4.7)	<0.001	2.8 (1.9, 4.9)	2.8 (1.8, 5.0)	0.564
Length of hospital stay, days	7.7 (4.6, 13.7)	6.6 (3.7, 11.6)	<0.001	7.6 (4.5, 12.8)	7.6 (4.6, 13.0)	0.093
ICU-free day, days	19.4 ± 10.2	21.6 ± 8.9	<0.001	20.1 ± 9.9	20.7 ± 9.5	0.069
Vasopressor-free day, days	22.2 ± 10.8	24.4 ± 9.0	<0.001	22.9 ± 10.4	23.6 ± 9.7	0.055

Note: Continuous variables are presented as median (interquartile), and categorical variables as count (%).

Abbreviations: ACE, acetaminophen; ICU, intensive care unit.

### Primary outcome

**FIGURE 2:**
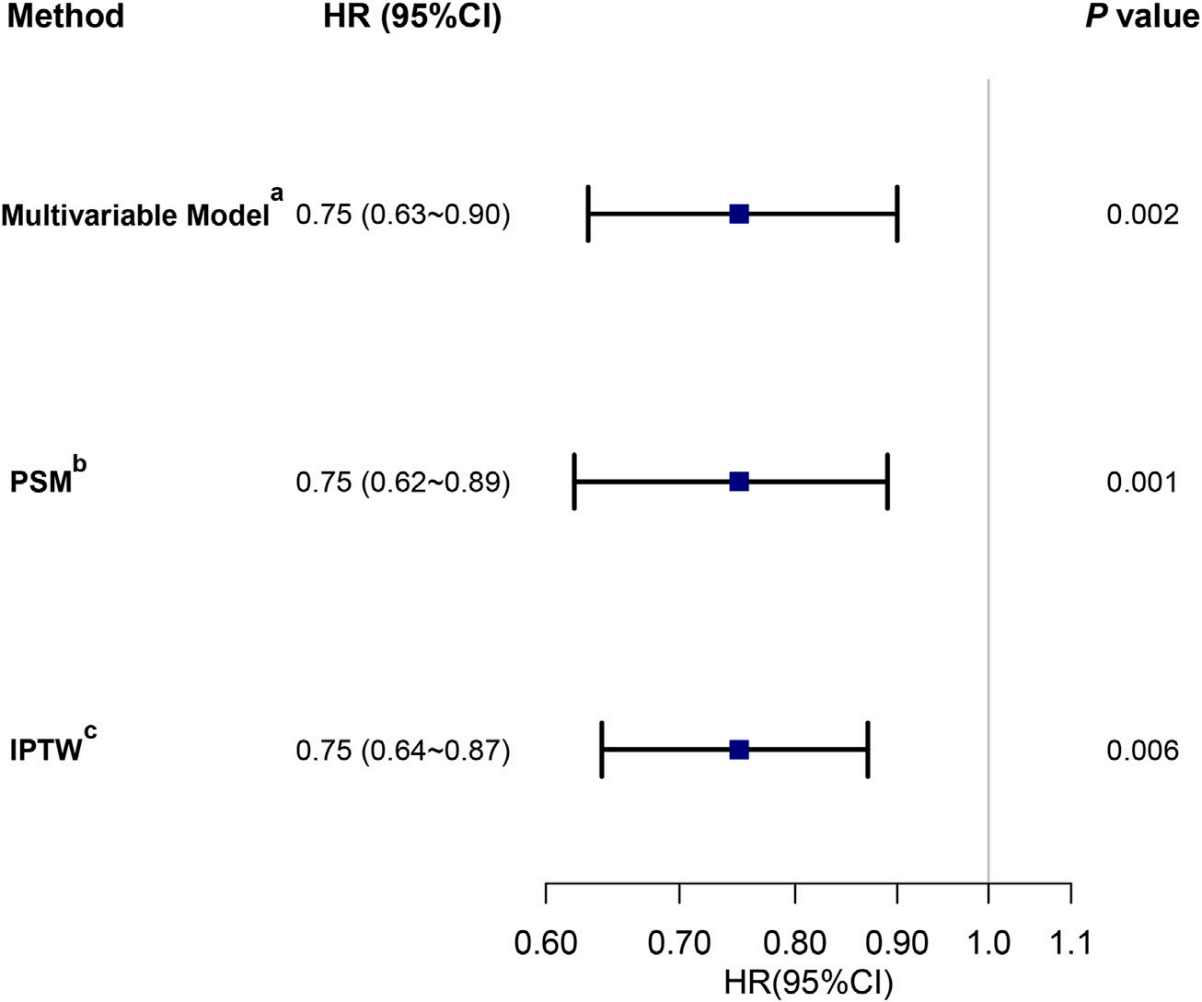
The association between acetaminophen exposure in the first 48 h after ICU admission and in-hospital mortality. Note: three different methods were used to address the association: (a) multivariable Cox regression analysis adjusted for all covariates (Table 1); (b) HR from a multivariable Cox regression with the same covariates, with additional adjustment for the propensity score; (c) HR from a multivariable Cox regression with the same covariates, with IPTW based on the propensity score. Abbreviations: HR, hazard ratio; CI, confidence interval; PSM, propensity score matching; IPTW, inverse probability of treatment weighting.

**FIGURE 3:**
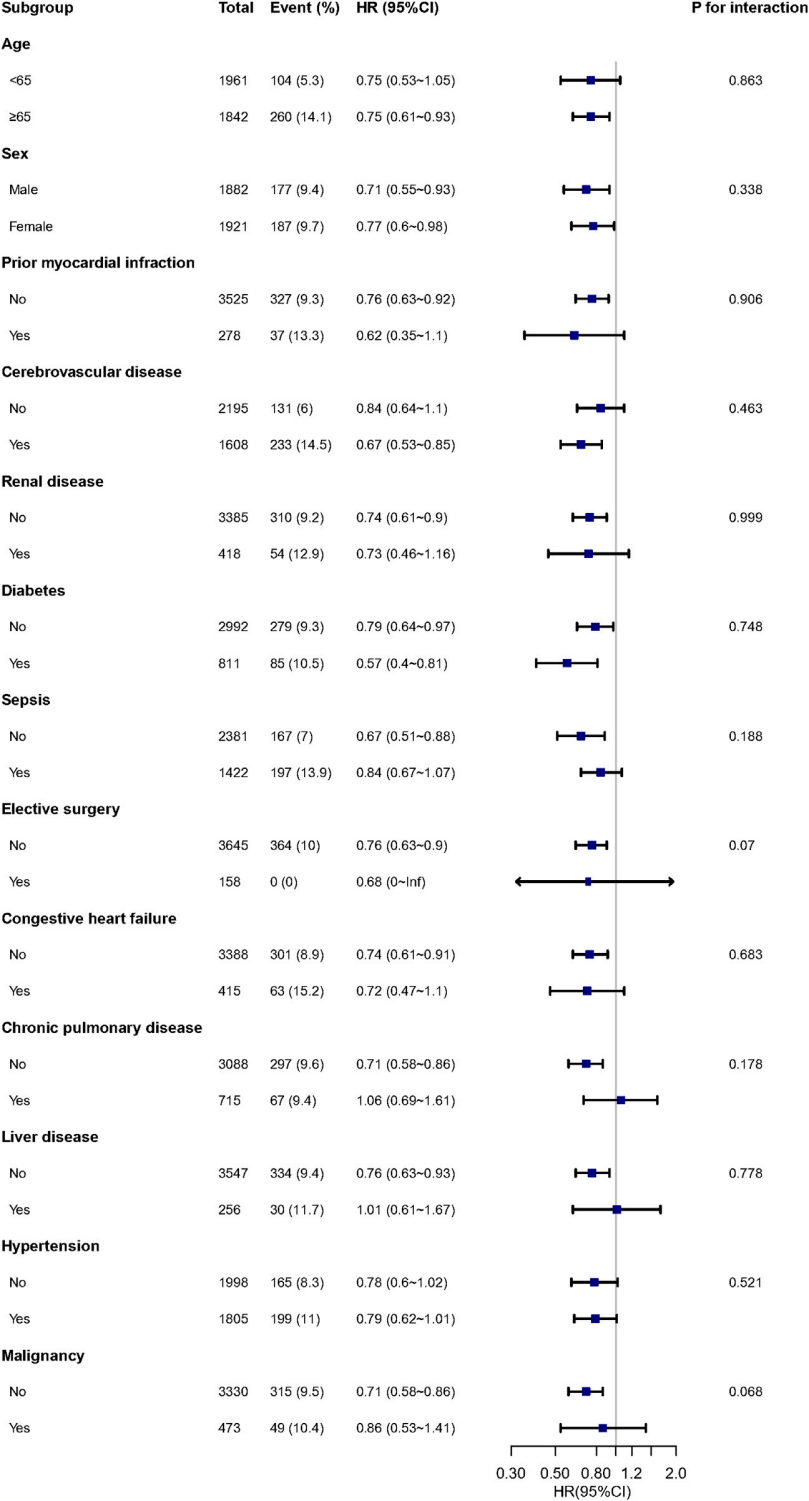
Subgroup and interaction analyses for in-hospital mortality. Note: Each stratification adjusted for all covariates in Table 1, except for the stratification factor itself. Abbreviations: HR, hazard ratio; CI, confidence interval.

The multivariable Cox regression revealed a reduced in-hospital mortality with acetaminophen use (Figure 2), exhibiting an adjusted HR of 0.75 (95% CI, 0.63–0.90, *P* = 0.002). Details of the regression are in Supplementary Table S2. Utilizing PSM and IPTW minimized covariate imbalances between the ACE and non-ACE groups, maintaining a consistent association (Figure 2). Subgroup analyses, considering factors such as age, sex, and histories of myocardial infarction, cerebrovascular disease, renal disease, diabetes, sepsis, elective surgery, congestive heart failure, chronic pulmonary disease, liver disease, hypertension, and malignancy, further supported this consistency across subgroups (Figure 3).

### Secondary outcomes with propensity score-matched cohorts

**FIGURE 4:**
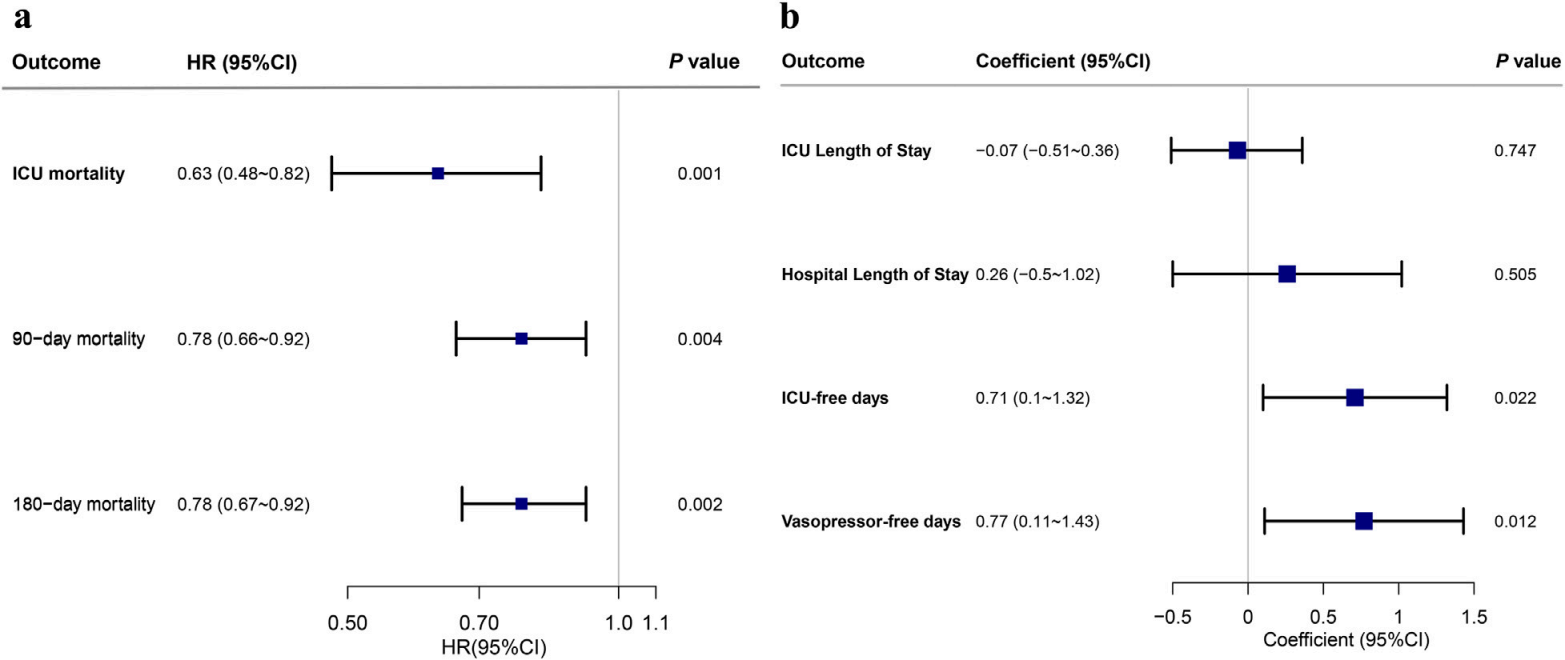
Acetaminophen exposure in the first 48 h after ICU admission and secondary outcomes in the matched cohort. Note: **(a)** HR and 95% CI from the multivariable Cox regression for ICU mortality, 90-day mortality, and 180-mortality. **(b)** Coefficient and 95% CI from the multivariable linear regression for ICU length of stay, hospital length of stay, ICU-free days to day 28, and vasopressor-free days to day 28. Abbreviations: HR, hazard ratio; CI, confidence interval; ICU, intensive care unit.

In the matched cohort, early administration of acetaminophen significantly reduced ICU mortality (HR, 0.63; 95% CI, 0.48–0.82; *P* = 0.001), 90-day mortality (HR, 0.78; 95% CI, 0.66–0.92; *P* = 0.004), and 180-day mortality (HR, 0.78; 95% CI, 0.67–0.92; *P* = 0.002) (Figure 4A). After adjusting for covariates, there were no significant associations between acetaminophen exposure and durations of ICU or hospital stays (Figure 4B), while early administration of acetaminophen was associated with longer ICU-free days to day 28 (*β* = 0.71, *P* = 0.022) and vasopressor-free days to day 28 (*β* = 0.77, *P* = 0.012) (Figure 4B).

### Sensitivity analyses

To evaluate the impact of missing data and multiple imputation on the primary outcome, multivariable Cox regression analyses were performed pre- and post-imputation. The findings remained consistent after multiple imputations for variables of interest (Supplementary Table S3).

## Discussion

Our study indicated that early acetaminophen use was associated with reduced in - hospital mortality in critically ill patients admitted to the SICU. The association was consistent across subgroup analyses and stable in sensitivity analyses, confirming the reliability of the result. Early acetaminophen administration was associated with lower ICU, 90-day, and 180-day mortality and with longer ICU-free days to day 28 and vasopressor-free days to day 28.

Research on the effects of acetaminophen in the SICU is limited; several prior studies involving critically ill patients have reported similar findings. Suzuki et al. (2015) conducted a multicenter retrospective observational study involving 15,818 critically ill patients and found that acetaminophen administration was associated with reduced in-hospital mortality. Similarly, Sun et al. (2024) reported that early acetaminophen use was associated with a lower risk of mortality in patients with sepsis. Several studies have reported that early postoperative acetaminophen administration was associated with a reduced risk of AKI in adults recovering from cardiac surgery (Xiong et al., 2023; Young et al., 2024). The study by Yi et al. (2024) indicated that acetaminophen was effective in reducing in-hospital mortality among critically ill individuals with gout and hypertension. These studies, like ours, highlight the potential protective effects of acetaminophen in critical illness, possibly due to its antioxidant properties that mitigate oxidative stress and inflammation.

However, the existing body of research also encompasses alternative perspectives, with certain studies yielding divergent results. Sakkat et al. performed a meta-analysis on 13 randomized controlled trials with 1,963 patients and discovered no notable difference in mortality between those treated with antipyretic therapy and those given a placebo among non-neurocritical ill patients (Sakkat et al., 2021). Holgersson and colleagues performed a meta-analysis on 42 trials with 5,140 participants, concluding that fever therapy appears to have no impact on the risk of death or serious adverse events (Holgersson et al., 2022). Xiong et al. found that early postoperative acetaminophen use associated with AKI after cardiac surgery but found no relationship between acetaminophen exposure with in-hospital mortality (Xiong et al., 2023). These contrasting results may be attributed to differences in study design, patient populations, the inclusion of different types of antipyretic drugs and the specific interventions used. For instance, meta-analyses included patients with a broader range of critical illnesses (Sakkat et al., 2021; Holgersson et al., 2022), while others focused specifically on cardiac surgery patients (Xiong et al., 2023). Additionally, the timing and dosage of acetaminophen administration may vary across studies, which could influence the observed outcomes. Despite these differences, our study adds to the growing body of evidence suggesting that early acetaminophen administration may have a beneficial impact on mortality in certain patient populations. Further prospective studies are needed to confirm these findings and to explore the underlying mechanisms.

Surgical patients, particularly those in the ICU, frequently experience significant oxidative stress and inflammatory damage following their procedures. Oxidative stress and systemic inflammation have been identified as key factors influencing postoperative outcomes (Prasain et al., 2013; Lopez et al., 2024; Zivkovic et al., 2024). The beneficial effects of early acetaminophen administration in SICU patients may be attributed to its antioxidant and anti - inflammatory properties.

Acetaminophen has been shown to inhibit hemoprotein - catalyzed lipid peroxidation, a process that can lead to cell membrane damage and subsequent organ dysfunction (Boutaud et al., 2010). In a rat model of rhabdomyolysis - induced kidney injury, acetaminophen significantly decreased markers of lipid peroxidation and preserved kidney function (Boutaud et al., 2010). This suggests that acetaminophen may protect against oxidative stress - induced tissue damage, which is a common feature in critically ill surgical patients. Moreover, acetaminophen may reduce the levels of cell - free hemoglobin, a byproduct of hemolysis that can cause oxidative stress and inflammation (Janz et al., 2013). Elevated levels of cell - free hemoglobin have been shown to be associated with increased mortality and acute kidney injury in patients with sepsis (Janz et al., 2013). By attenuating the harmful effects of cell - free hemoglobin, acetaminophen may help to mitigate the severity of organ dysfunction and improve patient outcomes. Additionally, acetaminophen has been shown to have anti - inflammatory effects. Acetaminophen has been shown to reduce the production of pro - inflammatory cytokines and enzymes, such as cyclooxygenase - 2 (COX - 2) and the inflammatory transcription factor - nuclear factor kappa-B (NF-κB), thereby diminishing the inflammatory response (Zhao et al., 2017). Previous research has largely demonstrated that acetaminophen exerts an ameliorating influence on sepsis - related outcomes (Janz et al., 2013; Janz et al., 2015; Sun et al., 2024). While sepsis does not afflict all patients in the SICU, every patient in this setting is characterized by a heightened inflammatory state and is susceptible to oxidative injury. Consequently, insights derived from sepsis models may shed light on the underlying mechanisms relevant to SICU patients.

Our analysis found no significant association between acetaminophen use and hospital length of stay. However, early acetaminophen administration was associated with longer ICU-free days to day 28 and vasopressor-free days to day 28. A shorter ICU stay might be due to either prompt recovery or early - stage death (Cho et al., 2021). Hospital resources and treatment decisions are some of the factors that can affect ICU stay duration, but they may not have a direct connection with patient prognosis. As a result, the accuracy of ICU stay as a prognostic indicator is somewhat reduced. Our findings on the benefits of early acetaminophen administration in terms of longer ICU - free days and vasopressor - free days to day 28 highlight its potential to improve clinical outcomes.

In summary, the antioxidant and anti - inflammatory properties of acetaminophen may explain its association with reduced mortality in SICU patients. By reducing oxidative stress and inflammation, early acetaminophen administration may help to protect against organ dysfunction and improve patient outcomes. Further prospective studies are needed to confirm these findings and to explore the optimal dosing regimens and timing of acetaminophen administration in this patient population.

The strengths of our study include its relatively large population-based cohort design from a representative database, which provides sufficient statistical power to examine the association between acetaminophen use and in-hospital mortality. Moreover, this study addresses the topic within the context of the SICU population, adding significant novelty and practical clinical relevance to the findings, which have considerable potential for real - world application.

Nonetheless, it is imperative to recognize certain limitations inherent in this study. Primarily, the study was unable to evaluate acetaminophen dosing due to insufficient detail in the registries employed, necessitating the treatment of exposure as a binary variable. The association between acetaminophen use and mortality may be influenced by dosage variations, where low doses might be inadequate for preventing mortality, while high doses could pose risks of hepatic and renal toxicity. Future prospective studies with precisely defined dosing regimens are needed to address this limitation. Second, the MIMIC database lacks specific information regarding the indications for acetaminophen administration, although it was most likely utilized primarily for analgesia and antipyresis. Third, as an observational study, we cannot definitively establish a causal relationship between acetaminophen use and the risk of in-hospital mortality among the study population. Despite our efforts using PSM, IPTW, and multivariable analyses to adjust for all relevant potential confounders, it remains conceivable that some unmeasured or unrecognized residual confounders (e.g., socioeconomic status or other environmental factors) may exist, potentially leading to an overestimation of the observed associations. Forth, the findings may not be broadly generalizable to diverse populations, as they are derived from one healthcare setting. Last, this study did not examine negative effects of acetaminophen use, like asthma and liver failure. Considering these limitations, it is essential to conduct well-designed multicenter controlled trials that specifically address the safety concerns associated with acetaminophen use and its dose-effect relationship in improving prognosis for patients in the SICU.

## Conclusion

This study indicated that acetaminophen administration within the initial 48 h following ICU admission was correlated with decreased in-hospital mortality among patients admitted to the SICU. The implications of these findings necessitate further investigation through prospective studies.

## Data Availability

Publicly available datasets were analyzed in this study. This data can be found here: https://mimic.mit.edu/.
