# Supplementary material for: Association of early acetaminophen administration with mortality in surgical intensive care patients: a retrospective cohort study
Source: Front Pharmacol. 2025 Aug 25;16:1588978. doi: 10.3389/fphar.2025.1588978 (PMC12414977; doi:10.3389/fphar.2025.1588978)
Supplement: Supplementary file 1 [file Supplementaryfile1.docx]

**Table S1. Percentage of missing data in the variables of interest.**

| **Variable** | **Missing rate (%)**  **(n = 5,474)** |
| --- | --- |
| **Baseline characteristics** |  |
| Age, years | 0% |
| Sex, female | 0% |
| Race | 0% |
| White | 0% |
| Black | 0% |
| Others/unknown | 0% |
| Weight, Kg | 1.85% |
| Admission following elective surgery | 0% |
| **Comorbidities** |  |
| Prior myocardial infarction | 0% |
| Congestive heart failure | 0% |
| Cerebrovascular disease | 0% |
| Chronic pulmonary disease | 0% |
| Renal disease | 0% |
| Liver disease | 0% |
| Hypertension | 0% |
| Diabetes | 0% |
| Sepsis | 0% |
| Malignant cancer | 0% |
| **Severity of illness** |  |
| SAPS II | 0% |
| Charlson comorbidity index | 0% |
| SOFA | 0% |
| OASIS | 0% |
| **Treatment** |  |
| Vasopressor | 0% |
| Renal replacement therapy | 0% |
| **Vital sign** |  |
| Mean blood pressure, mmHg | 0.26% |
| Respiratory rate, breaths/min | 0.24% |
| Temperature, °C | 0.84% |
| Heart rate, beats/min | 0.22% |
| **Laboratory findings** |  |
| WBC, K/µL | 0.37% |
| Platelets, K/µL | 0.38% |
| Hemoglobin, g/dL | 0.37% |
| Glucose, mg/dL | 0.97% |
| Creatinine, mg/dl | 0.29% |
| **Outcomes** |  |
| In-hospital mortality | 0% |
| ICU mortality | 0% |
| 90-day mortality | 0% |
| 180-day mortality | 0% |
| Length of hospital stay, days | 0% |
| Length of ICU stay, days | 0% |
| ICU-free-days to day 28 | 0% |
| Vasopressor-free-days to day 28 | 0% |

Abbreviations: SAPS, simplified acute physiology score; SOFA, sequential organ failure assessment; OASIS, oxford acute severity of illness score; WBC, white blood cell; ICU, intensive care unit.

**Table S2. Full multivariable model assessing impact of acetaminophen exposure on in-hospital mortality.**

| **Variable** | **Crude**  **HR (95%CI)** | **Crude *P* value** | **Adjusted**  **HR^a^ (95%CI)** | **Adjusted *P* value** |
| --- | --- | --- | --- | --- |
| Acetaminophen | 0.69 (0.59~0.81) | <0.001 | 0.75 (0.63~0.9) | 0.002 |
| Age | 1.03 (1.03~1.04) | <0.001 | 1.02 (1.01~1.03) | <0.001 |
| Sex |  |  |  |  |
| Male | ref. |  | ref. |  |
| Female | 1.28 (1.1~1.5) | 0.002 | 1.04 (0.87~1.24) | 0.651 |
| Race |  |  |  |  |
| White | ref. |  | ref. |  |
| Black | 0.89 (0.64~1.22) | 0.462 | 0.95 (0.68~1.33) | 0.779 |
| Others/unknown | 1.85 (1.57~2.19) | <0.001 | 1.6 (1.34~1.9) | <0.001 |
| Weight | 0.99 (0.99~0.99) | <0.001 | 1 (0.99~1) | 0.071 |
| Admission following elective surgery | 0.08 (0.01~0.56) | 0.011 | 0.26 (0.04~1.83) | 0.175 |
| Prior myocardial infarction | 1.33 (1.03~1.72) | 0.031 | 0.87 (0.67~1.15) | 0.338 |
| Congestive heart failure | 1.5 (1.23~1.83) | <0.001 | 1.14 (0.91~1.42) | 0.252 |
| Cerebrovascular disease | 2.19 (1.87~2.57) | <0.001 | 2.59 (2.13~3.16) | <0.001 |
| Chronic pulmonary disease | 0.94 (0.77~1.15) | 0.554 | 0.88 (0.71~1.1) | 0.261 |
| Renal disease | 1.2 (0.97~1.49) | 0.093 | 0.87 (0.64~1.18) | 0.367 |
| Liver disease | 1 (0.81~1.22) | 0.964 | 1.47 (1.13~1.93) | 0.005 |
| Hypertension | 1.18 (1.01~1.38) | 0.037 | 0.88 (0.73~1.06) | 0.175 |
| Diabetes | 1.02 (0.85~1.23) | 0.808 | 0.75 (0.6~0.93) | 0.008 |
| Sepsis | 1.02 (0.86~1.2) | 0.855 | 0.47 (0.39~0.57) | <0.001 |
| Malignant cancer | 0.91 (0.72~1.13) | 0.381 | 0.91 (0.68~1.22) | 0.51 |
| SAPS II | 1.04 (1.04~1.05) | <0.001 | 1.02 (1.02~1.03) | <0.001 |
| Charlson comorbidity index | 1.11 (1.09~1.14) | <0.001 | 0.99 (0.94~1.05) | 0.709 |
| SOFA | 1.08 (1.05~1.11) | <0.001 | 1.04 (1~1.08) | 0.043 |
| OASIS | 1.08 (1.07~1.08) | <0.001 | 1.04 (1.03~1.06) | <0.001 |
| Vasopressor | 1.87 (1.58~2.21) | <0.001 | 1.2 (0.99~1.46) | 0.07 |
| Renal replacement therapy | 1.18 (0.83~1.67) | 0.366 | 0.46 (0.29~0.71) | 0.001 |
| Mean blood pressure | 0.98 (0.98~0.98) | <0.001 | 0.99 (0.99~1) | 0.007 |
| Respiratory rate | 1.02 (1.01~1.03) | <0.001 | 1 (0.98~1.01) | 0.474 |
| Temperature | 1.13 (1.01~1.26) | 0.03 | 0.97 (0.86~1.09) | 0.614 |
| Heart rate, beats/min | 1.01 (1.01~1.01) | <0.001 | 1.01 (1~1.01) | 0.004 |
| WBC | 1.01 (1.01~1.01) | <0.001 | 1.01 (1~1.01) | 0.001 |
| Platelets | 1 (1~1) | 0.005 | 1 (1~1) | 0.813 |
| Hemoglobin | 0.95 (0.92~0.98) | 0.003 | 0.99 (0.95~1.03) | 0.71 |
| Glucose | 1.01 (1.01~1.01) | <0.001 | 1.01 (1.01~1.01) | <0.001 |
| Creatinine | 1.05 (1.01~1.09) | 0.008 | 1.08 (1.01~1.15) | 0.024 |

Note: ^a^ HR from the multivariable Cox proportional model adjusted for all covariates (Table 1).

Abbreviations: HR, Hazard ratio; CI, confidence interval; SAPS, simplified acute physiology score; SOFA, sequential organ failure assessment; OASIS, oxford acute severity of illness score; WBC, white blood cell.

**Table S3. Full multivariable model assessing impact of acetaminophen exposure on in-hospital mortality before and after multiple imputation.**

|  | **Before imputation** | | **After imputation** | |
| --- | --- | --- | --- | --- |
| **Variable** | **Adjuted**  **HR(95%CI)** | ***P* value** | **Adjusted**  **HR^a^ (95%CI)** | ***P* value** |
| Acetaminophen | 0.75 (0.62~0.9) | 0.002 | 0.75 (0.63~0.9) | 0.002 |
| Age | 1.02 (1.01~1.02) | <0.001 | 1.02 (1.01~1.03) | <0.001 |
| Sex |  |  |  |  |
| Male | ref. |  | ref. |  |
| Female | 1.05 (0.87~1.27) | 0.597 | 1.04 (0.87~1.24) | 0.651 |
| Race |  |  |  |  |
| White | ref. |  | ref. |  |
| Black | 1.05 (0.74~1.47) | 0.796 | 0.95 (0.68~1.33) | 0.779 |
| Others/unknown | 1.53 (1.27~1.84) | <0.001 | 1.6 (1.34~1.9) | <0.001 |
| Weight | 1 (0.99~1) | 0.068 | 1 (0.99~1) | 0.071 |
| Admission following elective surgery | 0.28 (0.04~2.03) | 0.21 | 0.26 (0.04~1.83) | 0.175 |
| Comorbidities |  |  |  |  |
| Prior myocardial infarction | 0.85 (0.64~1.13) | 0.268 | 0.87 (0.67~1.15) | 0.338 |
| Congestive heart failure | 1.14 (0.9~1.44) | 0.275 | 1.14 (0.91~1.42) | 0.252 |
| Cerebrovascular disease | 2.79 (2.26~3.45) | <0.001 | 2.59 (2.13~3.16) | <0.001 |
| Chronic pulmonary disease | 0.87 (0.69~1.1) | 0.25 | 0.88 (0.71~1.1) | 0.261 |
| Renal disease | 0.91 (0.66~1.26) | 0.567 | 0.87 (0.64~1.18) | 0.367 |
| Liver disease | 1.5 (1.13~1.98) | 0.005 | 1.47 (1.13~1.93) | 0.005 |
| Hypertension | 0.94 (0.77~1.15) | 0.526 | 0.88 (0.73~1.06) | 0.175 |
| Diabetes | 0.78 (0.62~0.98) | 0.032 | 0.75 (0.6~0.93) | 0.008 |
| Sepsis | 0.51 (0.41~0.62) | <0.001 | 0.47 (0.39~0.57) | <0.001 |
| Malignant cancer | 0.91 (0.67~1.25) | 0.576 | 0.91 (0.68~1.22) | 0.51 |
| Severity of illness |  |  |  |  |
| SAPS II | 1.02 (1.01~1.03) | <0.001 | 1.02 (1.02~1.03) | <0.001 |
| Charlson comorbidity index | 1 (0.95~1.06) | 0.931 | 0.99 (0.94~1.05) | 0.709 |
| SOFA | 1.05 (1~1.09) | 0.029 | 1.04 (1~1.08) | 0.043 |
| OASIS | 1.05 (1.03~1.06) | <0.001 | 1.04 (1.03~1.06) | <0.001 |
| Treatment |  |  |  |  |
| Vasopressor | 1.27 (1.03~1.55) | 0.025 | 1.2 (0.99~1.46) | 0.07 |
| Renal replacement therapy | 0.4 (0.25~0.64) | <0.001 | 0.46 (0.29~0.71) | 0.001 |
| Vital sign |  |  |  |  |
| Mean blood pressure | 0.99 (0.99~1) | 0.013 | 0.99 (0.99~1) | 0.007 |
| Respiratory rate | 1 (0.98~1.01) | 0.614 | 1 (0.98~1.01) | 0.474 |
| Temperature | 1 (0.89~1.13) | 0.986 | 0.97 (0.86~1.09) | 0.614 |
| Heart rate, beats/min | 1 (1~1.01) | 0.05 | 1.01 (1~1.01) | 0.004 |
| Laboratory findings |  |  |  |  |
| WBC | 1.02 (1.01~1.02) | <0.001 | 1.01 (1~1.01) | 0.001 |
| Platelets | 1 (1~1) | 0.965 | 1 (1~1) | 0.813 |
| Hemoglobin | 1 (0.95~1.04) | 0.85 | 0.99 (0.95~1.03) | 0.71 |
| Glucose | 1.01 (1~1.01) | <0.001 | 1.01 (1.01~1.01) | <0.001 |
| Creatinine | 1.07 (1~1.15) | 0.042 | 1.08 (1.01~1.15) | 0.024 |

Note: ^a^ HR from the multivariable Cox proportional model adjusted for all covariates (Table 1).

Abbreviations: HR, Hazard ratio; CI, confidence interval; SAPS, simplified acute physiology score; SOFA, sequential organ failure assessment; OASIS, oxford acute severity of illness score; WBC, white blood cell.
